# Supplementary material for: Zongertinib, a Novel HER2 Tyrosine Kinase Inhibitor, Maintains an Anticancer Activity for Trastuzumab Deruxtecan-Resistant Cancers Harboring HER2-Overexpression
Source: Int J Mol Sci. 2025 Oct 29;26(21):10515. doi: 10.3390/ijms262110515 (PMC12608020; doi:10.3390/ijms262110515)
Supplement: Supplementary file 1 [file ijms-26-10515-s001.zip › Supplementary Figure S1.pdf]

Fig S1

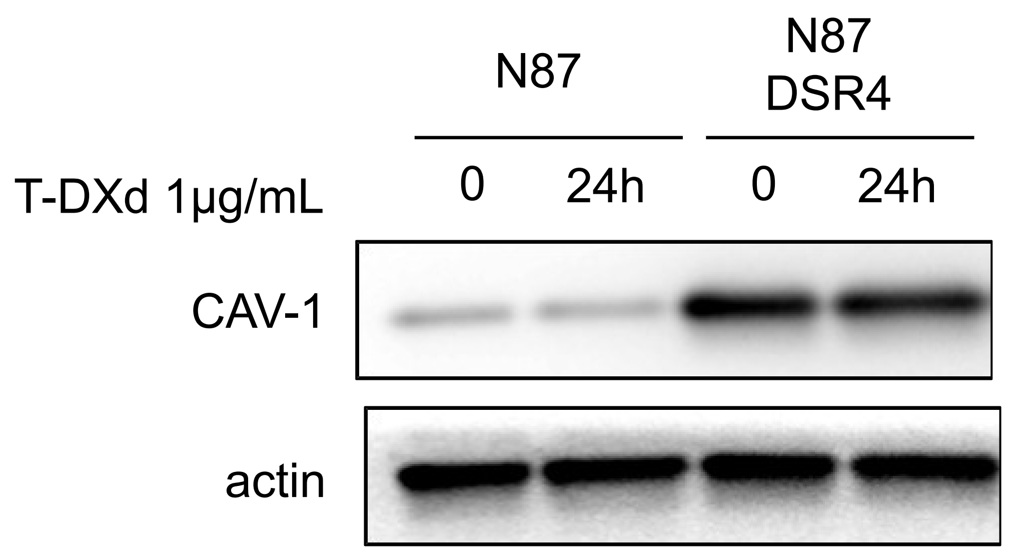

Either N87 or DSR4 cells were treated with T-DXd (1 μg/mL) or vehicle for indicated times. Indicated proteins were detected by Western blotting.
