# Supplementary material for: Zongertinib, a Novel HER2 Tyrosine Kinase Inhibitor, Maintains an Anticancer Activity for Trastuzumab Deruxtecan-Resistant Cancers Harboring HER2-Overexpression
Source: Int J Mol Sci. 2025 Oct 29;26(21):10515. doi: 10.3390/ijms262110515 (PMC12608020; doi:10.3390/ijms262110515)
Supplement: Supplementary file 1 [file ijms-26-10515-s001.zip › Supplementary Table S1.pdf]

Table S1

Mutation	Allele frequency (%)	
	N87	DSR4
HER2 S413L	14%	13%
HER2 F425L	16%	15%
HER2 L436V	17%	15%

Single nucleotide variants in HER2 gene and their allele frequency in N87 and DSR4 cells
